# Characterization of *Chlorella vulgaris* (Trebouxiophyceae) associated microbial communities^1^

**DOI:** 10.1111/jpy.13026

**Published:** 2020-06-22

**Authors:** Iris Haberkorn, Jean‐Claude Walser, Harald Helisch, Lukas Böcker, Stefan Belz, Markus Schuppler, Stefanos Fasoulas, Alexander Mathys

**Affiliations:** ^1^ Laboratory of Sustainable Food Processing Institute of Food, Nutrition and Health Swiss Federal Institute of Technology (ETH) Schmelzbergstrasse 9 8092 Zürich Switzerland; ^2^ Genetic Diversity Centre Swiss Federal Institute of Technology (ETH) Universitätsstrasse 16 8092 Zürich Switzerland; ^3^ Institute of Space System Engineering University of Stuttgart Pfaffenwaldring 29 70569 Stuttgart Germany; ^4^ Laboratory of Food Microbiology Institute of Food, Nutrition and Health Swiss Federal Institute of Technology (ETH) Schmelzbergstrasse 7 8092 Zürich Switzerland

**Keywords:** amplicon sequencing, bacterial diversity, *Chlorella vulgaris*, co‐culture, eukaryotic diversity

## Abstract

Microalgae exhibit extensive potential for counteracting imminent challenges in the nutraceutical, pharmaceutical, and biomaterial sectors, but lack economic viability. Biotechnological systems for contamination control could advance the economic viability of microalgal feedstock, but the selection of suitable strains that sustainably promote microalgal productivity remains challenging. In this study, total diversity in phototrophic *Chlorella vulgaris* cultures was assessed by amplicon sequencing comparing cultures subjected to five different cultivation conditions. Overall, 12 eukaryotic and 53 prokaryotic taxa were identified; Alphaproteobacteria (36.7%) dominated the prokaryotic and *C. vulgaris* (97.2%) the eukaryotic community. Despite altering cultivation conditions, 2 eukaryotic and 40 prokaryotic taxa remained stably associated with *C. vulgaris*; diversity between systems did not significantly differ (*P *> 0.05). Among those, 20 cultivable taxa were isolated and identified by 16S rDNA sequencing. Subsequently, controlled co‐cultures were investigated showing stable associations of *C. vulgaris* with *Sphingopyxis* sp. and *Pseudomonas* sp.. Out‐competition of *C. vulgaris* due to ammonium or phosphate limitation was not observed, despite significantly elevated growth of *Sphingopyxis* sp. and *Tistrella* sp.. (*P* < 0.05). Nevertheless, *C. vulgaris* growth was impaired by *Tistrella* sp.. Hence, the study provides a selection of stable indigenous prokaryotes and eukaryotes for artificially tailoring microbial biocenoses. Following a bottom‐up approach, it provides a base for controlled co‐cultures and thus the establishment of even more complex biocenoses using interkingdom assemblages. Such assemblages can benefit from functional richness for improved nutrient utilization, as well as bacterial load control, which can enhance microalgal feedstock production through improved culture stability and productivity.

**Abbreviations**
BB: biobench flat plate airlift photobioreactor
BHI: brain heart infusion
CfU: colony forming units
DSN: diluted seawater nitrogen medium
FPA: flat panel airlift photobioreactor
NGS: next‐generation sequencing
OTUs: operational taxonomic units
SI: shaking incubator
TOC: total organic carbon
TSA: tryptic soy agar
ZOTUs: zero‐radius operational taxonomic units

A projected world population growth to approximately 9.7 billion people by 2050 as well as environmental concerns necessitate the development of novel and more sustainable sources for the bio‐based industry (United Nations 2019). In that context, microalgae have attracted attention as they are highly efficient producers of feedstock for the biofuels, nutraceuticals, or pharmaceutical sectors, offering production concepts that are potentially more sustainable than those of other currently exploited resources (Henchion et al. 2017, Caporgno and Mathys 2018, Chaudhary et al. 2018). However, the competitiveness of microalgae with other plant‐based resources or chemically‐synthesized compounds is hampered, as cultivation costs for microalgae remain relatively high (Enzing et al. 2014). The primary bottlenecks to upstream microalgal operations were, for instance, attributed to impaired microalgal productivities resulting from culture contamination, which can lead to substantial biomass and thus economic losses. Such biomass losses were reported to amount up to 30% of the projected annual production volume for industrially relevant open pond systems (Enzing et al. 2014, Newby et al. 2016).

To avoid economic losses and render microalgae into viable feedstock, the development of contamination control strategies for microalgal crop protection is crucial, as monocultures are tedious to maintain in industrially‐relevant cultivation systems, due to their susceptibility to extraneous invasion by contaminants or grazers (Wijffels et al. 2010, Newby et al. 2016). Commonly exploited contamination control strategies in industrially‐relevant systems are based on hurdles, such as pH alterations or chemical treatments but are costly or can negatively impact microalgal cells or the environment (Wang et al. 2013, Newby et al. 2016). Biotechnological systems employing artificially tailored microbial biocenoses can advance microalgae feedstock production as a hurdle in the contamination control for industrially‐relevant microalgae upstream operations, due to their potential to improve culture stability while not bearing the aforementioned negative impacts (Le Chevanton et al. 2013, Zhang et al. 2018). Given a selection of appropriate strains, artificially tailored microbial biocenoses could even be exploited to elevate microalgal productivity (Cho et al. 2015). Additionally, biotechnological systems employing artificially tailored microbial biocenoses that rely on stable associations can foster the development of emerging processing technologies, such as pulsed electric field treatments aiming at a selective inactivation of prokaryotes within microalgal cultures. Currently, the advancement of such technologies is hampered by a lack in understanding eukaryotic and prokaryotic consortia, as well as underlying interactions, which, for instance, results in unknown target organisms (Buchmann et al. 2018, Buchmann and Mathys 2019). The identification of strains to tailor artificial microbial biocenoses as biotechnological systems for contamination control is key. Microbial diversity is not a guarantee for elevated productivity or improved culture stability. Microbial biocenoses benefiting from functional richness were shown to be more influential on productivity than those premised on species richness, providing enhanced microalgal stability or enhanced productivity through optimized resource utilization within a given niche (Su et al. 2015, Newby et al. 2016, Toyama et al. 2018). For instance, Rhizobiales were shown to be involved in nitrogen fixation; additionally, the delivery of phytohormones and B‐vitamins are among possible interactions and were described to have a positive impact on microalgal growth (Krohn‐Molt et al. 2013).

Harnessing biotechnological systems for microalgal crop protection in a controlled way requires investigating community structures and understanding underlying interactions from naturally occurring systems as an important first step for assembling artificially tailored microbial biocenoses to efficiently improve microalgal feedstock production (Zhang et al. 2018). In industrially relevant cultivation systems, the microbiome associated with microalgae was shown to undergo fluctuations and adapt to changing external conditions, such as differences in light or nutrient regimes (Eigemann et al. 2013, Fulbright et al. 2018). Identifying a stable microalgae–associated microbiome despite changing external conditions can aid in identifying strains for assembling artificial microbial biocenoses that promote culture stability in a sustainable manner. Employing stably associated strains with desired functional traits for assembling tailored biocenoses would reduce the necessity of adapting the crop protection strategy to specific operational upstream conditions and thereby simplify industrial application. Additionally, stable microalgae–associated microbiomes within different environments might result from mutual adaptations based on functional relations and optimized niche packing for improved nutrient utilization. Hence, bacterial strains were isolated from *Chlorella vulgaris* cultures, characterized by 16S rDNA sequencing, and based on potential functional traits selected to characterize interactions in co‐culture with *C. vulgaris*.

Next‐generation sequencing (NGS)‐based analysis approaches provide a sensitive tool for investigating complex microbial communities. Culture‐independent high‐throughput sequencing methods can be used for complementing culture‐dependent methods (Al‐Awadhi et al. 2013). Although culture‐dependent methods have been reported to only capture a fraction of the microbial diversity in a system, they provide a tool for assessing the physiology of isolates and constructing databases for metagenomics studies (Stefani et al. 2015).

The study characterized prokaryotic and eukaryotic microbial community structures in phototrophic *Chlorella vulgaris* SAG 211‐12 cultures, subjected to different cultivation conditions, including three different reactor systems and five cultivation conditions employing NGS‐based sequencing of the V3‐V4 and V4 hypervariable region of the prokaryotic 16S and eukaryotic 18S rDNA, respectively. The study aimed to identify strains that remain stable in *C. vulgaris* cultures despite altering external conditions. Following a bottom‐up approach, controlled co‐cultures with selected bacterial strains were conducted focusing on stable *C. vulgaris* cultures to provide a base for the establishment of even more complex biocenoses to sustainably promote microalgal growth.

## MATERIALS AND METHODS

### Cultivation of *Chlorella vulgaris* for microbiome analysis

Axenic *Chlorella vulgaris* SAG 211‐12 was obtained originally from the culture collection of algae at Goettingen University, Germany. A pre‐culture was maintained in sterile diluted seawater nitrogen (DSN) medium in 1 L culture volume using a 1 L Schott flask (Pohl et al. 1987). Cultures were illuminated continuously, applying 60 µmol of photons · m^−2^ · s^−1^ as a mean photosynthetically active photon flux density (PPFD; blue‐red LED panels), continuously aerated using pressurized air at 2.5 l_n_ · min^−1^ (500 ppm CO_2_) to provide mixing, and kept at temperatures between 25 and 26°C at the Institute of Space Systems, University of Stuttgart, Germany (Bretschneider et al. 2016). The pre‐culture was maintained for 1 year by serial sub‐culture, where at the end of the exponential growth phase cultures were inoculated to a dry weight (DW) of 0.2–0.3 g · L^−1^ using fresh DSN medium, which was inferred from a linear fit for optical density (OD) at 750 nm and *C. vulgaris* DW, as described elsewhere (Helisch et al. 2016). Owing the laboratory setup, pre‐cultures were handled non‐sterile. All associated reasearch activities have been conducted for assisting and accompanying an experiment onboard the International Space Station intended to investigate the capability and potential of photobioreactors for future bioregenerative life support systems (Helisch et al. 2020).

Subsequently, phototrophic *Chlorella vulgaris* cultures were conducted in three different reactor systems and five cultivation conditions. Samples obtained from flasks cultures in a shaking incubator (SI) and a biobench flat plate PBR (BB), both cultivated at 25°C and 30°C under sterile conditions, as well as a flat panel airlift PBR (FPA) under non‐sterile conditions were compared.

For SI cultures, *Chlorella vulgaris* was inoculated to an initial DW of 0.2–0.3 g · L^−1^ as 600 mL cultures using DSN medium in 1 L Erlenmeyer flasks (Pohl et al. 1987). Experiments were conducted at 25 ± 0.2°C or 30 ± 0.2°C and a continuous illumination applying a mean PPFD of 240 µmol photons · m^−2^ · s^−1^ (Multitron Pro; Infors AG, Bottmingen, Switzerland). For BB cultures, *C. vulgaris* was inoculated to an initial DW of 0.2–0.3 g · L^−1^ as 1.8 L cultures using DSN medium in sterile 2.0 L cultivation chambers (Biostream International BV, Doetinchem, Netherlands). Experiments were conducted at 25 ± 0.2°C or 30 ± 0.2°C, constant aeration (2 l_n_ · min^−1^) with ambient CO_2_ (400 ppm) and a continuous illumination applying a mean PPFD of 240 µmol photons · m^−2^ · s^−1^. Cultivations in FPA reactor systems followed a procedure described by Helisch et al. (2016). Briefly, *C. vulgaris* cultures were inoculated to an initial DW of 0.2–0.3 g · L^−1^ in DSN medium and cultivated in 6.0 L cultivation chambers at 26 ± 1°C, continuous illumination applying a mean PPFD of 350 µmol photons · m^−2^ s^−1^, and 8% v/v CO_2_ (Subitec, Stuttgart, Germany).

Ammonium (NH_4_^+^) and phosphate (PO_4_^3‐^) content were monitored daily using control strips (Hach Lange GmbH, Germany) and maintained at 300 mg · L^−1^ and 200 mg · L^−1^, respectively, by supplementing cultures with the corresponding volume using ammonium ([NH_4_^+^] = 120 mg · mL^−1^) and phosphate ([PO_4_^3‐^] = 100 mg · mL^−1^) stock solutions. Cultivations were conducted in biological duplicates each lasting for 14 d. Samples were retrieved from the pre‐culture, on days 0, 1, 4, 8, 11, and 15 after inoculation for BB and SI cultures, and on days 0, 4, 8, and 15 for FPA cultures.

### Genomic DNA extraction and library preparation

A pellet of 20 mL of culture was harvested by centrifugation at 16,000*g* for 5 min. Until further analysis, samples were stored at –20°C. Total genomic DNA was extracted using a FastDNA^®^ SPIN Kit for Soil (MP Biomedicals, Illkirch, France). Concentration and purity of the extracted DNA were determined (NanoDrop ND 1000 Spectrophotometer; Thermo Fisher Scientific Inc., Waltham, MA, USA) and genomic DNA concentration was normalized in molecular grade water (1.25 ng · µL^−1^).

Library preparation followed a two‐step PCR protocol. Limited‐cycle PCR was conducted in 25 µL reaction volume using KAPA HiFi HotStart ReadyMix 2x (12.5 µL) (Kapa Biosystems, Wilmington, MA, USA), template DNA (5 µL), forward and reverse primers (0.3 mM each), and molecular grade water. All primers were modified to include a hexanucleotide barcode and Illumina (Illumina Inc., San Diego, CA, USA) adapters. The target region on the 18S rDNA was amplified with the primer pair TAReuk454FWD1 (5’‐CCA GCA SCY GCG GTA ATT CC‐3’) and TAReukREV3 (5’‐ACT TTC GTT CTT GAT YRA‐3’; Stoeck et al. 2010), following thermocycling conditions with an initial denaturation at 95°C for 300 s, 10 cycles at 98°C for 20 s, 57°C for 15 s, 72°C for 15 s, followed by 33 cycles at 98°C for 20 s, 47°C for 15 s, and 72°C for 15 s, and a final elongation at 72°C for 60 s (Stoeck et al. 2010). The prokaryotic V3‐V4 hypervariable region was amplified using the primer pair 341F (5’‐CCT ACG GGN GGC WGC AG‐3’) and 806R (5’‐GGA CTA CNV GGG TWT CTA AT‐3’; Hugerth et al. 2014, Apprill et al. 2015). PCR conditions were modified from Hugerth et al. (2014) and included an initial denaturation at 95°C for 300 s, 1 cycle of 98°C for 60 s, 23 cycles of 98°C for 20 s, 51°C for 20 s, and 72°C for 12 s, and a final extension at 72°C for 120 s. Negative controls were run replacing template DNA by molecular grade water. Positive controls for eukaryotic community analysis were run using DNA of axenic *Chlorella vulgaris* SAG 211‐12 extracted applying the above described protocol.

Triplicate PCR reactions were carried out for each sample and target region. PCR products from identical samples were pooled, cleaned using solid‐phase reversible immobilization beads (Genomic Diversity Center, GDC; ETH Zürich, Switzerland), and used as a template for index PCR to attach dual indices using the Illumina Nextera XT index kit v2 (Illumina Inc., San Diego, CA, USA).

Index PCR included limited‐cycle PCR product (2 µL), KAPA HiFi HotStart ReadyMix 2x (10 µL), molecular grade water (4 µL), and Nextera indexing primers (2 µL). Thermocycling conditions of the PCR included an initial denaturation at 95°C for 180 s, followed by 10 cycles at 95°C for 30 s, 55°C for 30 s, and 72°C for 30 s, and a final extension at 72°C for 300 s. Index PCR products were cleaned and DNA concentration was determined by a Qubit dsDNA BR Assay (Thermo Fisher Scientific Inc., Waltham, MA, USA) on a Spark 10M microplate reader (Tecan Trading AG, Männedorf, Switzerland). Concentration of genomic DNA was normalized (3 nM) and 5 µL were used for pooling the libraries. Concentration and purity of the pooled library were controlled by a Qubit dsDNA HR Assay on a Spark 10M microplate reader and High Sensitivity D1000 kit on a TapeStation (2200 TapeStation; Agilent Technologies, Santa Clara, CA, USA), respectively. Paired‐end sequencing was performed using 20 pM of the prepared library in a single MiSeq 2 × 300 bp flow cell line spiked with 20% of PhiX (Illumina Inc., San Diego, CA, USA), according to the manufacturer’s protocol.

### 16S rDNA sequencing of bacterial isolates

Serial dilutions of the fresh microalgal suspension (10^−1^–10^−5^) were prepared in sterile maximum recovery diluent (BD DIFCO^TM^, Franklin Lakes, NJ, USA) in triplicates and each dilution was spread (100 µL) onto brain heart infusion (BHI) agar plates (Oxoid^TM^, Thermo Fisher Scientific Inc.). The plates were incubated in an SI using the same conditions as those used for *Chlorella vulgaris* cultivation. Morphologically different bacterial colonies were isolated on fresh BHI plates and incubated (30°C, 2 d).

The DNA of bacterial isolates was extracted using a GenElute Bacterial Genomic DNA kit (Sigma‐Aldrich Co. LLC., St. Louis, MO, USA), according to the manufacturer's instructions. Purity and concentration of isolated DNA were determined spectrophotometrically (NanoDrop ND 1000 Spectrophotometer; Thermo Fisher Scientific Inc.). The extracted DNA was normalized (2.5‐5 ng · µL^−1^) in molecular grade water and nearly complete 16S rDNA amplified using the primer pair 27F (5’‐AGAGTTTGATCNTGGCTCAG‐3’) and 1492R (5’‐TACGGYTACCTTGTTACG‐3’; Lane 1991). PCR reactions were carried out in 20 µL reaction volumes using PCR Master Mix (2X) (10 µL; Thermo Fisher Scientific Inc.), forward and reverse primers (0.5 µM each), dH_2_O (7 µL), and genomic DNA (1 µL). Thermocycling conditions were 95°C for 120 s, 32 cycles of 95°C for 30 s, 55°C for 30 s, 72°C for 90 s, and 72°C for 10 min. GelRed‐stained agarose gels (1.5%) were used to visualize PCR products, cleaning and Sanger sequencing of PCR products were conducted externally at Microsynth AG (Balgach, Switzerland).

### Bioinformatics analysis of amplicon sequencing data

Initial quality control of raw reads was conducted in FastQC (v.0.11.2). Subsequent bioinformatics analysis included trimming, quality filtering, and clustering steps. A typical pipeline in 16S amplicon analysis includes the generation of operational taxonomic units (OTUs), which are defined as a cluster of reads with a 97% agreement in pairwise sequence alignment. Based on that clustering, a representative sequence is selected for annotation; consequently, all sequences within an OTU are assigned the same annotation (Nguyen et al. 2016). However, clusters might be defective, as, for instance, PCR and sequencing can introduce errors, such as point errors or PCR chimeras, which make the obtained reads difficult to distinguish from true biological variation (Edgar 2016b). To account for those errors, Illumina de‐noisers such as UNOISE were developed for inferring accurate biological template sequences from erroneous reads by first employing a de‐noising step to remove point errors and then a filtering of chimeric amplicons yielding zero‐radius OTUs (ZOTUs). Therefore, ZOTUs are valid OTUs providing higher biological resolution. Taxonomic assignments of prokaryotic ZOTUs were predicted using SINTAX (v.10.0.240) with Silva as reference database (Edgar 2016a). Eukaryotic taxonomic assignment followed a procedure suggested by Deiner et al. (2016).

Data evaluation and statistical analysis were conducted in R version 3.5.0. Rare ZOTUs with less than 10 total counts and samples with less than 100 counts were excluded from analysis. To evaluate prokaryotic community composition, different measures of alpha‐diversity, covering aspects of evenness, diversity, and abundance were applied. A chi‐square test was used to analyze differences in Shannon diversity to put equal weight on rare and abundant species for diversity analysis (Morris et al. 2014).

### Co‐cultures

Axenic *Chlorella vulgaris* SAG 211‐12 was obtained from the culture collection of algae at the University of Goettingen, Germany. *C. vulgaris* was inoculated into sterile DSN medium to 5 × 10^6^ cells · mL^−1^ 48 h prior to the experiment to obtain cells in the exponential growth phase. The axenic culture was cultivated in a shaking incubator (Multitron Pro, Infors AG) at 25°C, 70% rH, 150 rpm, and 7% v/v CO_2_ and continuous illumination with a mean PPFD of 36 μmol photons · m^−2^ · s^−1^. Subsequently, the *C. vulgaris* suspension was centrifuged at 1,200*g* for 10 min (Hermle Labortechnik GmbH, Wehingen, Germany) and the pellet was diluted with DSN medium to a cell concentration of 10^7^ cells · mL^−1^.

*Tistrella mobilis* TH‐33 (KF783213.1), *Pseudomonas pseudoalcaligenes* CLR9 (KF478199.1), and *Sphingopyxis* sp. AX‐A (JQ418293.1) were grown on tryptic soy agar (TSA) plates (3.0% tryptic soy broth [TSB] in dH_2_O, 1.5% agar) at 30°C. For the co‐cultivation experiment, single colonies were isolated and incubated in liquid TSB medium (3.0% TSB in dH_2_O) at 30°C and 150 rpm for 12 h (Multitron Pro, Infors AG, Switzerland). Subsequently, the bacterial suspensions were diluted to a concentration of 10^5^ cells · mL^−1^ and 35 mL aliquots of the suspensions were centrifuged at 5,000*g* for 10 min (Hermle Labortechnik GmbH). The supernatants were discarded and the pellets were washed three times by suspending them in sterile DSN medium and centrifuging at 5,000*g* for 10 min to remove all remaining TSB (Hermle Labortechnik GmbH).

For the co‐cultures, axenic *Chlorella vulgaris* was inoculated to 10^7^ cells · mL^−1^ in 35 mL cultivation volume using 100 mL Erlenmeyer flasks and mixed with the bacterial pellets (10^5^ cells · mL^−1^). The samples were cultivated in a shaking incubator (Multitron Pro, Infors AG) at 25°C, 70% rH, 150 rpm, and 7% v/v CO_2_ and continuous illumination with a mean PPFD of 36 μmol photons · m^−2^ · s^−1^. All cultivations were conducted as biological triplicates, including controls of axenic *C. vulgaris* and bacterial cultures in sterile DSN applying the same conditions as described above.

### Cultivation monitoring

Monitoring of *Chlorella vulgaris* growth was carried out for each biological replicate in duplicates by loading a Helber cell‐counting chamber (VWR International GmbH, Dietikon, Switzerland) with 2 µL of undiluted cell suspension and counting the cells using a DM6 light microscope at a magnification of 400 (Leica Microsystems GmbH, Heerbrugg, Switzerland). Additionally, the DW was determined by filtering 1 mL of each culture through dried and preweighed 1.2 µm filters (Whatman, GE Healthcare, Chicago, IL, USA). The biomass retentate was dried at 100°C for 24 h and weighed subsequently.

Growth of the bacterial strains in co‐cultivation with *C. vulgaris* was monitored by plate counts in triplicates for each biological replicate. Serial dilutions between 10^−1^ and 10^−6^ were prepared in maximum recovery diluent (BD DIFCO^TM^) and 25 µL of each dilution was pipetted onto TSB agar square plates (3% TSB and 1.5% agar in dH_2_O). The plates were incubated at 30°C and CfU were counted after 48 h.

Ammonia (NH_4_^+^) and phosphate (PO_4_^3‐^) content were monitored in the supernatant by centrifuging 1 mL of sample at 1,200*g* for 5 min, using test strips for ammonium and phosphate (Merck KGaA, Darmstadt, Germany). The pH of the samples was measured with a pH electrode (Metrohm AG, Herisau, Switzerland). Total organic carbon (TOC) content was analyzed using a SHIMADZU TOC‐V total organic carbon analyzer (Shimadzu Schweiz GmbH, Reinach BL, Switzerland). A volume of 8 mL of each replicate was centrifuged at 1,200*g* for 5 min (Hermle Labortechnik GmbH) and the supernatant was collected. The samples were filtered using a pressure filter system with 0.22 μm filter discs (Sartorius Lab Instruments GmbH & Co. KG, Goettingen, Germany) after adjusting the pH to 1 by adding 0.05 M hydrochloric acid (Sigma‐Aldrich Inc.). Data visualization for co‐cultures was done in OriginPro 2019; statistical analysis was conducted in R version 3.5.0. For the analysis of differences in *Chlorella vulgaris* growth between the co‐cultures and the axenic *C. vulgaris* cultures, Welch’s *t*‐test was used, due to unequal variances, at a significance level of 5%. For the analysis of differences between bacteria growth in the co‐cultures and the bacteria controls, a Wilcoxon rank‐sum test was carried out since the data was not normally distributed.

### Scanning electron microscopy

Scanning electron microscopy (SEM) was carried out at ScopeM for co‐cultures of *Chlorella vulgaris* with *Tistrella* sp., 48 h after inoculation. For the sample preparation, 2 mL of the *C. vulgaris* and *Tistrella* sp. co‐culture was fixed in a mixture of 0.5% glutaraldehyde and 2% formaldehyde followed by a staining with 1% osmium tetraoxide. After fixation, the sample was dehydrated in ethanol and critically point dried with CO_2_. For microscopic analysis, the samples were sputter coated with 6 nm platinum palladium in a compact coating unit (Safematic GmbH, Bad Ragaz, Switzerland) and analyzed with a Zeiss Leo1530 scanning electron microscope (Carl Zeiss AG, Oberkochen, Germany) at 2kV using an InLens detector.

### Data availability statement

The data sets generated and/or analyzed during the current study are available in the European Nucleotide Archive (ENA) repository under the accession number ERP111559 for amplicon associated data and ERP112294 for data obtained from sequencing of bacterial isolates.

## RESULTS

### Eukaryotic community

**Fig. 1:**
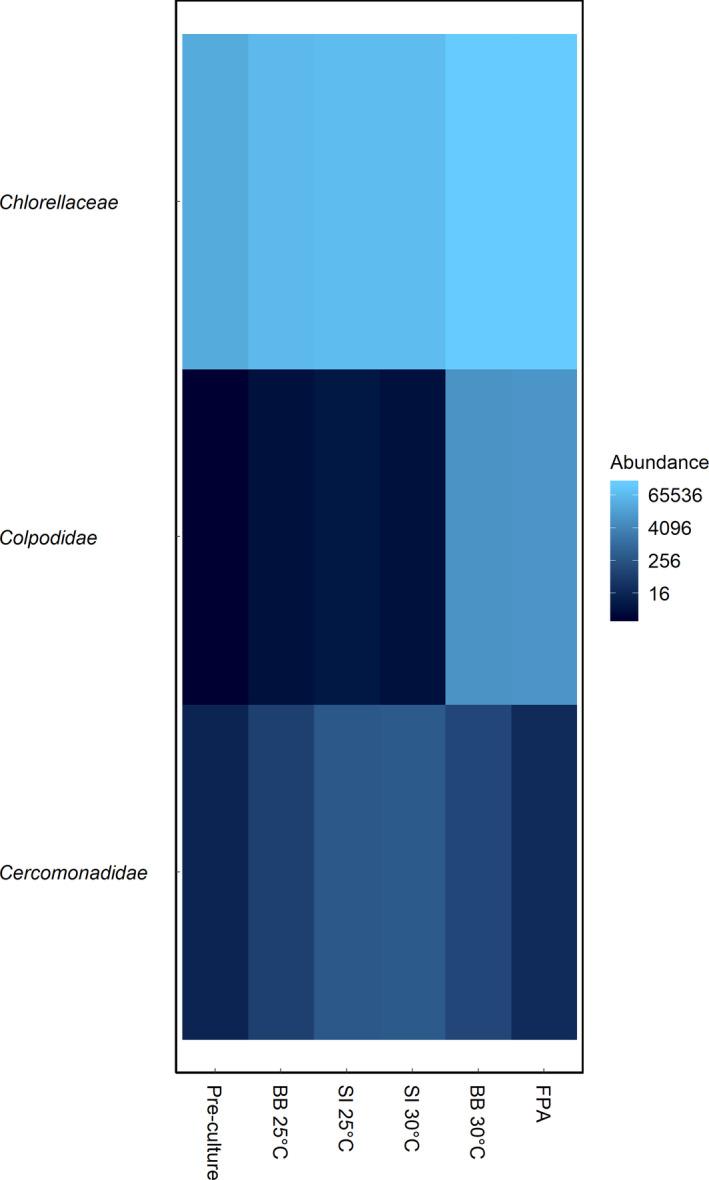
Heat map of eukaryotic community (family level) abundance (read counts) within *Chlorella vulgaris* cultures of samples obtained from flasks within a shaking incubator (SI) and biobench flat plate PBR (BB), flat panel airlift PBR (FPA) cultures, as well as the pre‐culture. BB and SI cultivations were conducted at 25 and 30°C. [Color figure can be viewed at wileyonlinelibrary.com]

Within the reactor systems investigated, amplicon sequencing of the 18S rDNA identified three eukaryotic taxa affiliated with *Chlorella* sp., *Paracercomonas* sp., and *Colpoda* sp. (Fig. 1). *C. vulgaris* dominated the eukaryotic community of each cultivation condition investigated, accounting for 99.9% of total sequence reads in the pre‐culture, 99.5% in SI, 95.9% in BB, and 94.3% in FPA samples. All reactor systems shared three ZOTUs affiliated with *Chlorella*, *Paracercomonas* sp., and *Colpoda* sp..

SI cultivations conducted at 25 and 30°C showed similar abundance levels of *Chlorella vulgaris* (99.5%) for both conditions, as well as for *Paracercomonas* sp. (0.5%) and *Colpoda* sp. with 0.008% and 0.006%, respectively. Comparing the two temperature regimes for BB cultivations showed higher *C. vulgaris* abundances in the 25°C cultures (99.9%) than in the 30°C cultures (94.4%), with *Colpoda* sp. reaching 5.2% abundance in the 30°C cultures compared to 0.006% in the 25°C cultures. In FPA cultures, *Colpoda* sp. and *Paracercomonas* sp. abundance amounted to 5.7% and 0.01%, respectively.

### Prokaryotic community

Prokaryotic diversity was investigated using two 16S rDNA sequencing‐based methods: amplicon sequencing of the whole microbiome using NGS‐based technology and sequencing of bacterial isolates. Overall, the approach resulted in 75 combined sequence variants representing the maximum possible diversity obtained by the two sequencing methods. Sequencing of bacterial isolates generated a total of 197 sequence variants. Subsequent primer trimming of those sequences, dereplication, and clustering at 97% identity level reduced the number of different sequence variants from bacterial isolate sequencing to 20 unique sequences. Cultivable strains characterized by Sanger sequencing of the 16S rDNA were affiliated with Sphingobacteriaceae, Comamonadaceae, Pseudomonadaceae, Rhodospirillaceae, Caulobacteraceae, Bradyrhizobiaceae, Sphingomonadaceae, and Phyllobacteriaceae. The sequences obtained from Sanger sequencing covered high‐ and low‐abundant taxa identified by amplicon sequencing, where the lowest abundant taxon identified by bacterial isolate sequencing was affiliated with *Achromobacter* sp. at an abundance level of 0.09% for the amplicon sequencing approach.

**Fig. 2:**
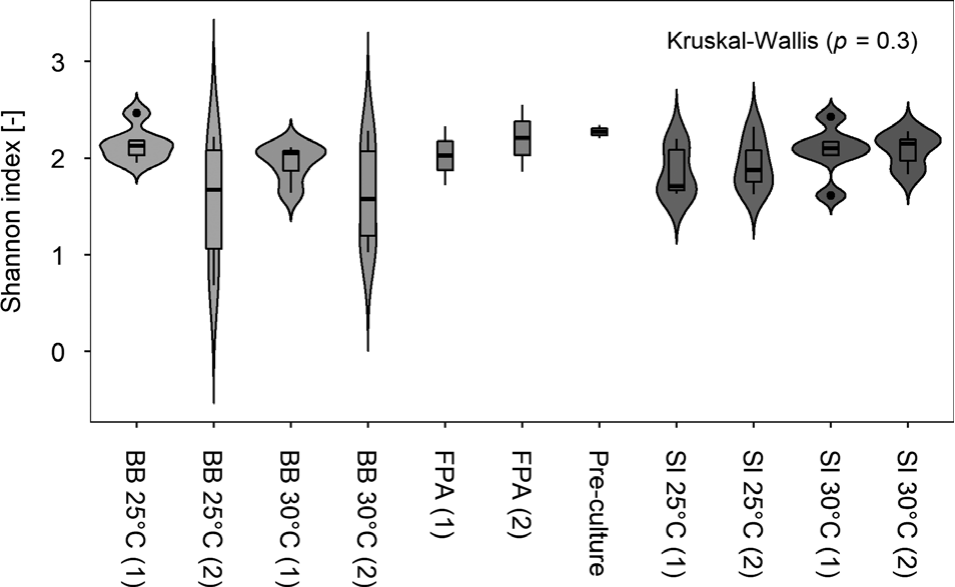
Violin plot of prokaryotic Shannon diversity. Data are provided for shaking incubator (SI) and biobench flat plate PBR (BB) samples cultivated at 25 and 30°C, as well as their pre‐culture and flat panel airlift PBR (FPA) samples for biological duplicate cultures. Statistical assessment was based on a global Kruskal–Wallis test (*P* < 0.05 indicates statistically significant difference).

The majority of read counts generated by 16S rDNA amplicon sequencing was eukaryotic (91.3%), whereas only 8.7% was prokaryotic. Moreover, amplicon sequencing identified 53 prokaryotic ZOTUs spanning 44 genera, 30 families, 19 orders, and 6 phyla (Proteobacteria (50.9%), Bacteroidetes (12.5%), Actinobacteria (1.4%), Gemmatimonadetes (1.1%), and Planctomycetes (0.01%)). Along with the prokaryotes, Chlorophyta (33.9%) were identified. Of the 53 ZOTUs identified by amplicon sequencing, 51, 50, and 43 were associated with BB, FPA, and SI cultivation systems, respectively. Among those, 40 ZOTUs were identified overlapping between all three cultivation systems. The cultivation conditions investigated did not significantly influence the prokaryotic diversity, as indicated by a non‐significant chi‐square test of the Shannon diversity index, which also showed that prokaryotic diversity was not significantly different between the biological replicates (chi‐square test, χ^2^_10_ = 11.85, *P >* 0.05; Fig. 2).

**Fig. 3:**
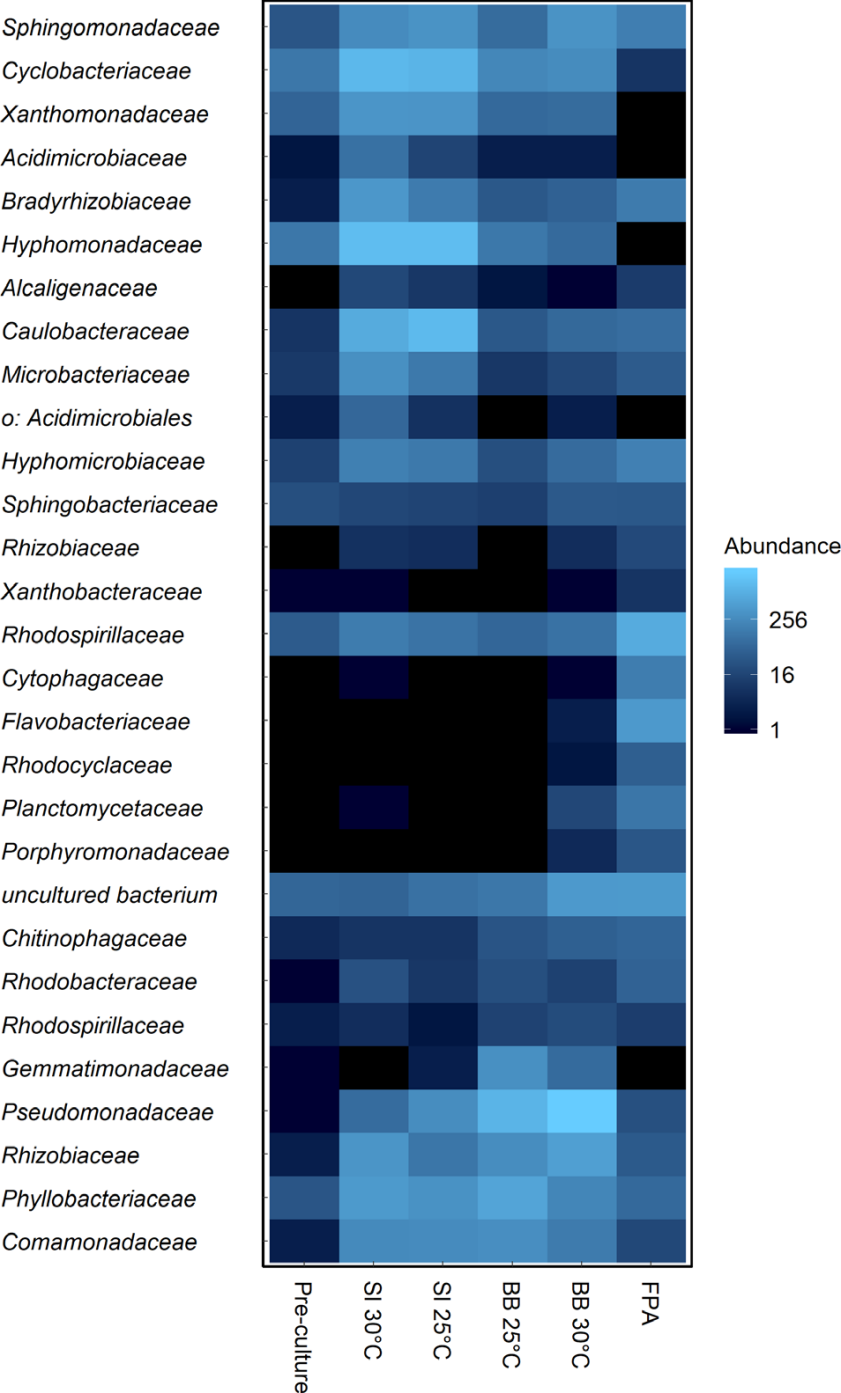
Heat map of prokaryotic community (family level) abundance (read counts) within *Chlorella vulgaris* cultures of samples obtained from flasks within a shaking incubator (SI) and biobench flat plate PBR (BB), flat panel airlift PBR (FPA) cultures, as well as the pre‐culture. BB and SI cultivations were conducted at 25 and 30°C. [Color figure can be viewed at wileyonlinelibrary.com]

Although prokaryotic diversity did not differ significantly between the cultivation systems investigated, a variation in abundance distribution was observed. In FPA, Rhodospirillaceae (26.8%), an uncultured bacterium (14.8%), and Rhizobiaceae (11.5%) were the most abundant prokaryotic families. In SI cultivation conducted at 25°C, Hyphomonadaceae (24.5%), Caulobacteraceae (21.4%), and Cyclobacteriaceae (17.7%) dominated; similarly, in the cultivations conducted at 30°C Hyphomonadaceae (23.1%), Cyclobacteriaceae (19.4%), and Caulobacteraceae (11%) dominated. For BB cultivations conducted at 25°C prokaryotes affiliated with Pseudomonadaceae (32.9%), Phyllobacteriaceae (17.8%), and Gemmatimonadaceae (8.6%) were most prevalent (Fig. 3). In BB cultivations conducted at 30°C also Pseudomonadaceae dominated (51%), but were followed by Rhizobiaceae (10.2%) and an uncultured bacterium (8.2%) as second and third most abundant prokaryotic family, respectively. Two ZOTUs were only identified for FPA cultures and were affiliated with the prokaryotic genera *Flavobacterium* (1.9%) and *Dyadobacter* (0.4%). In the BB and SI systems, the prokaryotic genera *Cecembia*, *Glycocaulis*, and a non‐assigned bacterium affiliated with the family of Acidimicrobiaceae were identified, but not in FPA samples. BB and FPA samples shared 8 ZOTUs that were not identified in the SI samples including the prokaryotic genera *Blastopirellula*, *Dysgonomonas*, *Aequorivita*, *Methyloversatilis*, *Azospirillum*, *Rhodopseudomonas*, *Sphingomonas*, and *Rhodobacter*.

### Co‐culture

Following a bottom‐up approach, controlled co‐cultures with selected bacterial strains were conducted focusing on stable *Chlorella vulgaris* cultures. Three cultivable prokaryotes were selected for controlled co‐cultivation studies with *C. vulgaris*. Criteria for selecting strains included the stable occurrence across all five investigated cultivation conditions, the ability to isolate the strains, and functional traits they might add to biocenoses. Stably occurring taxa included on the family level Chitinophagaceae, Sphingobacteriaceae, Comamonadaceae, Pseudomonadaceae, Rhodospirillaceae, Caulobacteraceae, Bradyrhizobiaceae, Sphingomonadaceae, Hyphomicrobiaceae, Phyllobacteriaceae. Among those, cultivable strains characterized by Sanger sequencing of the 16S rDNA were affiliated with Sphingobacteriaceae, Comamonadaceae, Pseudomonadaceae, Rhodospirillaceae, Caulobacteraceae, Bradyrhizobiaceae, Sphingomonadaceae, and Phyllobacteriaceae. Final strain selection for artificially tailored microbial biocenoses was then based on functional traits that could offer stability or enhance productivity of *C. vulgaris* through optimized resource utilization. On the species level, *Tistrella* sp. (Rhodospirillaceae), *Sphingopyxis* sp. (Sphingomonadaceae), and *Pseudomonas* sp. (Pseudomonadaceae) were selected to investigate their effect on *C. vulgaris* culture stability and productivity. Considering the non‐diazotrophic nature of microalgae, members affiliated with the order of Rhodospirillaceae, such as *Tistrella* sp. were selected, as they were shown of being involved in nitrogen fixation. Both, Rhodospirillaceae and Sphingomonadales such as *Sphingopyxis* sp. were suggested to be involved in plant growth promotion where previously a connection between plant growth promoting bacteria in the rhizosphere of plants and those promoting the growth of microalgae was suggested (Kim et al. 2014, Ramanan et al. 2015). Bacterial attenuation of photosynthetic oxygen tension within the proximate microalgal microenvironment was suggested for members affiliated with Pseudomonadaceae and thereby might contribute to the creation of optimal growth conditions (Mouget et al. 1995).

**Fig. 4:**
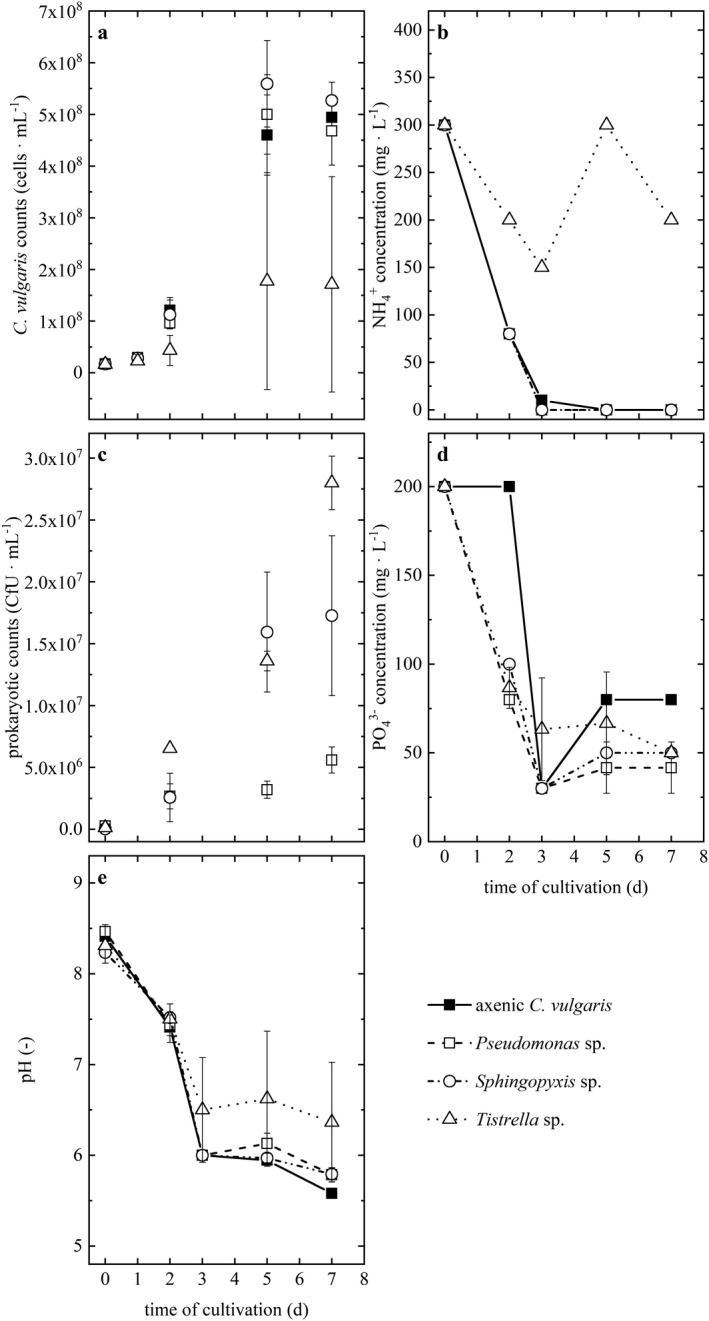
*Chlorella vulgaris* (cells · mL^−1^) (a) and prokaryotic (CfU · mL^−1^) cell counts (c), ammonium (NH^4+^) (b) and phosphate (PO_4_^3‐^) (d) concentration, as well as pH (e) development in the axenic *C. vulgaris* (■) and co‐cultures with *Pseudomonas* sp. (□), *Sphingopyxis* sp. (Ο), and *Tistrella* sp. (Δ), cultivated in a shaking incubator. Error bars denote the standard deviation of duplicate measurements from three biological replicates.

*Pseudomonas* sp. and *Sphingopyxis* sp. did not impair *Chlorella vulgaris* growth, compared to the axenic control (Fig. 4a). Maximum *C. vulgaris* biomass yields reached 5.0 × 10^8^ ± 7.7 × 10^7^ cells · mL^−1^ and 5.6 × 10^8^ ± 8.4 × 10^7^ cells · mL^−1^ in co‐culture with *Pseudomonas* sp. and *Sphingopyxis* sp., respectively, compared to 4.6 × 10^8^ ± 7.8 × 10^7^ cells · mL^−1^ obtained for axenic cultures on day 5 of cultivation. In contrast, in the co‐cultivation samples of *C. vulgaris* with *Tistrella* sp., attenuation of *C. vulgaris* growth was observed where biomass yields were significantly lower reaching 1.8 × 10^8^ ± 2.1 × 10^8^ cells · mL^−1^ on day 5 of cultivation compared to the axenic control (Welch’s *t*‐test, *t*_3_ = 10.713, *P* < 0.05).

*Sphingopyxis* sp. and *Tistrella* sp. growth was stimulated by the presence of *Chlorella vulgaris* (Fig. 4c). The concentration of *Sphingopyxis* sp. was significantly lower (Wilcoxon rank‐sum test, W = 27, *P* < 0.05) in the bacterial control culture (9.3 x 10^4^ CfU · mL^−1^) than in the co‐cultures (1.8 × 10^7^ CfU · mL^−1^) on day 5 of cultivation. *Tistrella* sp. in co‐cultivation with *C. vulgaris* showed an increase in CfU · mL^−1^ by 2.0 log_10_ from 1.3 × 10^5^ to 1.4 × 10^7^ CfU · mL^−1^ within the first 5 d of cultivation and was thus significantly higher than the *Tistrella* sp. concentration in the control (7.1 × 10^5^ CfU · mL^−1^; Wilcoxon rank‐sum test, W = 18, *P* < 0.05). *Pseudomonas* sp. in co‐culture with *C. vulgaris* showed an increase in CfU · mL^−1^ by 1.3 log_10_ within the first 2 days of cultivation to a concentration of 2.47 × 10^6^ CfU · mL^−1^, compared to a significantly lower concentration of 9.5 × 10^5^ CfU · mL^−1^ in the control (Wilcoxon rank‐sum test, W = 18, *P* < 0.05). However, on day 5 concentrations approached comparable CfU with 3.6 × 10^6^ and 2.4 × 10^6^ CfU for control and co‐culture samples (Wilcoxon rank‐sum test, W = 3, *P *> 0.05), respectively.

In co‐cultures of *Chlorella vulgaris* with *Sphingopyxis* sp. and *Pseudomonas* sp., ammonium was readily consumed within the first 72 h of cultivation (Fig. 4b). While phosphate consumption in *Tistrella* sp. co‐cultures was comparable to *Sphingopyxis* sp. and *Pseudomonas* sp. co‐cultures (Fig. 4d), less ammonium was consumed in co‐culture with *Tistrella* sp. with an attenuation on day 3. The development of pH was comparable for the axenic and co‐cultivation samples and decreased with cultivation time (Fig. 4e), which might relate to the generation of H^+^ ions following ammonium consumption (Grobbelaar 2013).

The TOC content in *Chlorella vulgaris* and *Tistrella* sp. co‐cultures on day 5 amounted to 98.4 ± 3.4 mg · L^−1^, compared to 77.0 ± 10.0 mg · L^−1^, 74.0 ± 5.0 mg · L^−1^, and 72.4 ± 5.6 mg · L^−1^ for co‐cultures with *Sphingopyxis* sp., axenic *C. vulgaris*, and co‐cultures with *Pseudomonas* sp., respectively.

**Fig. 5:**
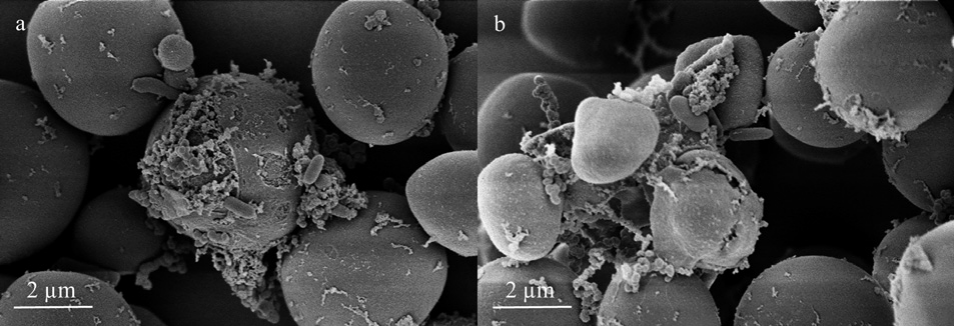
Representative SEM images of the *Chlorella vulgaris*–*Tistrella* sp. co‐culture after 48 h of cultivation.

SEM analysis showed *Tistrella* sp. cells to occur on the surface of *Chlorella vulgaris* cells and within aggregates of extracellular structures (Fig. 5, a and b). Some *C. vulgaris* cells exhibited rough surfaces and bacteria cells appeared to be attached to it (Fig. 5, a and b). Additionally, *C. vulgaris* cell envelopes were noticed to harbor bacteria cells and *C. vulgaris* appeared to break open, revealing intracellular structures. SEM pictures also showed *Tistrella* sp. closely attached to *C. vulgaris* cells losing outer membranous structures (Fig. 5a).

## DISCUSSION

Harnessing biotechnological systems based on artificially tailored microbial biocenoses can aid in advancing microalgal feedstock production in industrially relevant settings by improving culture stability. The identification of strains to construct artificial microbial biocenoses is key. Therefore, in a first step, the study aimed at characterizing total prokaryotic and eukaryotic microbial community structures in phototrophic *Chlorella vulgaris* cultures by amplicon sequencing.

The observed indigenous prokaryotic and eukaryotic taxa might have originated from the non‐sterile handling of the pre‐culture, leading to a fortuitous invasion of extraneous taxa over time during the 1‐year pre‐culture phase. Drivers for the selection of prokaryotes in microalgal cultures were, among others, associated with environmental conditions, strain specificity, and phylotype‐based assemblies (Ramanan et al. 2015). For instance, the employed sea‐salt–based DSN medium could have favored the assembly of the observed *C. vulgaris*–associated prokaryotic community profiles, as the obtained results are consistent with observations for marine ecosystems where Alphaproteobacteria are reported as the major prokaryotic fraction, followed by Gammaproteobacteria and Bacteroidetes (Stevens et al. 2005, Yang et al. 2016).

Previous studies have described using prokaryotic or polyalgal co‐cultures for fostering the stability of microalgae cultures, but the potential of tailoring microbial biocenoses using other microeukaryotes has been addressed to a lesser extend (Cho et al. 2015, 2017, Toyama et al. 2018). A study conducted by Cho et al. (2019) showed that the co‐cultivation of microalgae with other microeukaryotes, such as *Colpoda* sp. can decrease bacterial loads in *Chlorella vulgaris* cultures due to their bacterivorous behavior and therefore highlighted its potential as bacterial load control strategy. Although the study did not encompass an ecological scope, microeukaryotes, such as *Paracercomonas* sp. or *Colpoda* sp. might bear a yet unexploited potential for tailoring artificial microbial biocenoses as biological contamination control for microalgal crop protection (Smith and McBride 2014). Given their observed ability of remaining stably associated in the *C. vulgaris* cultures investigated, they show potential for sustainably promoting microalgal growth despite changing external conditions. Additionally, *Paracercomonas* sp. and *Colpoda* sp. were shown to exert bacterivorous behavior, which can aid in limiting bacterial loads (Bass et al. 2009, Cho et al. 2019). Furthermore, they might add functional traits to the niche supporting microalgal growth by nutrient supply. Heterotrophic flagellates, such as *Paracercomonas* sp. were shown of being capable to recover nutrients, mainly inorganic compounds and ammonia, from detritus and bacteria. Thereby, they enhanced the nutrient availability for other organisms, pivotal especially in oligotrophic environments (Plötner et al. 2014). Interestingly, *Colpoda* sp. is a microalgae predator and a major grazer of *C. vulgaris*. Although *Colpoda* sp. has been shown to be capable of causing culture crash in large‐scale *C. vulgaris* cultures within only short periods of time, no culture breakdown was observed in this study (Wang et al. 2017). The results are in accordance with Lakaniemi et al. (2012) who reported a coexistence of *C. vulgaris* and *Colpoda* sp. without any culture crash, where lytic activities of *Colpoda* sp. were related to desired processes, such as nutrient recycling (Lakaniemi et al. 2012, Carney et al. 2014, Wang et al. 2017). A steady state of predator–prey relationships was described as a continuous replacement of prey by its sustained reproduction (Habte and Alexander 1978). Continuous reproduction and moderate interactions (i.e., non‐erasing behavior) could assist maintaining a stable equilibrium among prokaryotes and eukaryotes in *C. vulgaris* cultures. Hence, using eukaryotes such as *Paracercomonas* sp. or *Colpoda* sp. for artificially tailoring microbial biocenoses could contribute to establishing microalgal crop protection strategies with sustained microalgal productivity by adding functional traits based on nutrient exchange, as well as bacterial load control. In accordance, read counts assigned to prokaryotes in the *C. vulgaris* cultures investigated in this study were low.

The prokaryotic diversity in the *Chlorella vulgaris* cultures investigated was rather limited to in total 53 ZOTUs, which is in accordance with existing literature (Krohn‐Molt et al. 2013). Low diversity was described to result from a mutual adaptation of the microbial community based on functional relations and optimized niche packing for improved nutrient utilization (Krohn‐Molt et al. 2017). Hence, species in a natural biocenoses characterized by a low diversity arise as promising candidates for an exploitation as biotechnological contamination control strategy, as they provide evidence for relying on mutual adaptation delivering desirable functional traits. Biocenoses relying on such interactions could advance microalgal culture stability through improved nutrient utilization.

Among the 53 prokaryotic ZOTUs identified, 40 ZOTUs remained stably associated in the *Chlorella vulgaris* cultures across the different cultivation conditions investigated. Hence, the obtained data indicated that certain species could get eradicated when cultured under specific conditions. The microbiome associated with microalgae can undergo fluctuations and adapt to external conditions, such as changing light or nutrient regimes (Eigemann et al. 2013, Fulbright et al. 2018). Fluctuating operational conditions, including the temperature and light regime, occur in industrially relevant cultivation systems. An eradication of prokaryotes can be a consequence from an adaptation due to unfavorable growth conditions or a colonization and subsequent out‐competition by other strains. Unfavorable growth conditions resulting in an eradication of prokaryotes might hold true for species that were unique for BB and FPA, but were not identified in SI, including on the genus level *Blastopirellula*, *Dysgonomonas*, *Aequorivita*, *Methyloversatilis*, *Azospirillum*, *Rhodopseudomonas*, *Sphingomonas*, and *Rhodobacter*. BB and FPA cultivation systems are, among other factors, characterized by different surface to volume ratios and therefore, for instance, differ in the light intensity applied to the cultures, which was shown to affect prokaryotic community structures in microalgae cultures (Zhang et al. 2019). Additionally, BB and FPA employ different mixing regimes based on turbulences created by aeration and thus distinct ways for gas introduction, which could have influenced the prokaryotic community structure. The aforementioned taxa were excluded as candidates for further co‐cultivation studies, as they did not persist across the cultivation conditions applied. The majority of the identified ZOTUs (40 of the identified 53 ZOTUs) remained stably associated across the cultivation conditions applied. These observations are in accordance with Lakaniemi et al. (2012), who reported no significant difference in prokaryotic diversity comparing *C. vulgaris* cultures subjected to different cultivations in photobioreactors. Closed bioreactors provide advantages over open pond systems as they allow a prevention of extraneous invasion by other species, thereby regulating contamination to low levels. Hence, the competitive pressure on the investigated microbiome was rather low and contributed to fostering a stable occurrence of the observed taxa. Future studies might focus on challenging the ecosystem dynamics by, for instance, investigating microbiome stability under open pond conditions to increase competitive pressure. Experiments harnessing defined contamination events, for instance, by introducing pathogens during cultivation could provide useful insights on the stability of the biocenoses or whether the microbiome can serve as a protective culture for sustaining *C. vulgaris* growth.

Harnessing prokaryotic species that remain stably associated with microalgae for artificially tailored microbial biocenoses targeting contamination control can reduce the necessity of adapting the crop protection strategy to specific operational upstream conditions and thereby simplify industrial applications. If resulting from a mutual adaptation for this specific niche, such prokaryotes could sustainably promote microalgal growth due to optimized functional relations required in a consortium with *Chlorella vulgaris*. Based on that assumption, three cultivable prokaryotes were selected for controlled co‐cultivation studies with *C. vulgaris*. Strain selection for artificially tailored microbial biocenoses should be based on functional traits for offering stability or enhancing productivity through optimized resource utilization, as diversity is not a guarantee for improved culture stability (Su et al. 2015, Newby et al. 2016). Optimal niche packing can elevate the amount of available resources, allowing for positive growth rates that make biocenoses less prone to extinction (Pellissier et al. 2018). Phosphorus, nitrogen, and carbon dioxide as carbon sources constitute the main macronutrients consumed by phototrophic *C. vulgaris* for biomass generation. On species level, *Tistrella* sp. (Rhodospirillaceae), *Sphingopyxis* sp. (Sphingomonadaceae), and *Pseudomonas* sp. (Pseudomonadaceae) were selected to investigate their effect on *C. vulgaris* culture stability and productivity.

Co‐cultures of *Chlorella vulgaris* with *Pseudomonas* sp. or *Sphingopyxis* sp. showed similar *C. vulgaris* productivity if compared to the axenic culture and thus did not cause culture breakdown but sustained *C. vulgaris* productivity. However, none of the cultures investigated resulted in a promotion of *C. vulgaris* productivity. Although Le Chevanton et al. (2013) showed that a co‐culture of *Dunaliella* with only one prokaryotic species can result into microalgal growth promotion, various studies reported employing multispecies assemblages to achieve positive effects on microalgal growth performance (Cho et al. 2015, Toyama et al. 2018). Multispecies assemblages benefit from a division of the metabolic pathways among the different consortia members, thereby alleviating the metabolic burden for a single species. These interactions were shown to result into higher biomass yields if compared to monocultures, but can be unpredictable and difficult to control (Zhang et al. 2018, Naidoo et al. 2019). Employing multispecies assemblages, instead of the two species cultures with *Sphingopyxis* sp. and *Pseudomonas* sp., could aid in enhancing *C. vulgaris* growth performance by optimizing nutrient utilization, if selecting species along an ecological gradient. This could improve nutrient exchange and foster productivity in addition to the observed culture stability. Bratbak and Thingstad (1985) suggested using more complex systems for improved culture stability and productivity, but additionally proposed including higher trophic levels for establishing interkingdom biocenoses that would not only allow for nutrient exchange, but also for bacterial load control. In this study, not only prokaryotes, but also the microeukaryotes *Colpoda* sp. and *Paracercomonas* sp. were identified of being stably associated with *C. vulgaris*. Biotechnological systems for contamination control harnessing functionally rich interkingdom assemblages of prokaryotes and microeukaryotes, as those identified in this study could provide potential for improving stability and productivity in microalgae cultures through improved nutrient utilization and bacterial load control.

Interestingly, *Sphingopyxis* sp. benefitted from the co‐culture with *Chlorella vulgaris* resulting in significantly increased CfU compared to the axenic control, while not hampering *C. vulgaris* productivity. Interactions between microalgae and bacteria can relate to an exchange of nutrients and growth impairment to a limitation therein (Ramanan et al. 2016). Phosphorus, nitrogen, and carbon dioxide as carbon sources constitute the macronutrients consumed by phototrophic *C. vulgaris* for biomass generation. According to the carbon limitation theory, heterotrophic bacteria rely on organic carbon as an energy source, which in co‐culture is supplied by microalgae. In return, microalgae depend on phosphorous and nitrogen provided by bacteria. In commensalistic relationships, bacteria control the availability of phosphorous for microalgae, while microalgae control the excretion of organic exudates in order to prevent their own out‐competition. During phosphate limitation though, it was shown that microalgae produce increased amounts of organic exudates (Currie and Kalff 1984, Bratbak and Thingstad 1985). Carbon and phosphate levels were comparable between the co‐culture and the axenic culture. From day 3 of cultivation, a limitation of ammonium and concomitantly a stagnation of *C. vulgaris* growth was observed, which was comparable to the axenic control. Hence, elevated *Sphingopyxis* sp. growth did not lead to an out‐competition of *C. vulgaris* due to a macronutrient limitation, as no phosphate limitation occurred in those co‐culture samples and ammonium consumption was comparable to the control, being depleted after 3 d of cultivation. In co‐culture with *Pseudomonas* sp., no growth promotion was observed, neither for *C. vulgaris* nor for *Pseudomonas* sp.. Based on the ammonium and phosphate consumption pattern, co‐culture did not lead to an out‐competition of either species due to macronutrient limitation, but no special benefit seemed to result from the interaction. By implication, neither *Sphingopyxis* sp. nor *Pseudomonas* sp. co‐cultures allowed to sustain *C. vulgaris* productivity during ammonium limitation, which might owe the fact that those are no nitrogen‐fixating strains. Interestingly, axenic cultures and co‐cultures with *Sphingopyxis* sp. and *Pseudomonas* sp. presented the same ammonium consumption rate, indicating that *C. vulgaris* was the main consumer of ammonium in those cultures and that co‐cultivation with prokaryotes did not lead to increased ammonium needs by the microalgae. Following ammonium depletion on day 3 of cultivation, growth retardation of *C. vulgaris* was observed initially on day 5. *Chlorella* was shown of being capable to sustain growth even under nitrogen limiting conditions with unaffected growth rates up to two weeks (Negi et al. 2016).

In contrast, impaired *Chlorella vulgaris* productivity was observed in co‐cultures with *Tistrella* sp. with stagnating growth from day 3 of cultivation. Growth impairment of *C. vulgaris* did not result from an out‐competition due to a limitation of the macronutrients nitrogen, phosphorus, or carbon. Neither ammonium nor phosphate was readily consumed in the co‐cultures and carbon dioxide was continuously provided. Hence, co‐culture with *Tistrella* sp. might have impaired *C. vulgaris* growth or even led to cell death due to a limitation in micronutrients or the generation of secondary, growth‐impairing metabolites by *Tistrella* sp. from day 3 of cultivation. In that context, an active killing of *C. vulgaris* by *Tistrella* sp. to obtain nutrients for sustained bacterial growth could have occurred. Bratbak and Thingstad (1985) proposed that bacteria feed on dead algae by growing on material, such as carbon, phosphorus, or nitrogen retained in microalgal cells, where bacteria in close attachment to dead microalgae provide an indication thereof. Accordingly, SEM analysis revealed *Tistrella* sp. in close association with *C. vulgaris*. Bacterial cells accumulated around dead cells or around those forming extracellular structures. SEM images also showed *Tistrella* sp. closely attached to *C. vulgaris* cells losing outer membranous structures. Concomitantly, increased levels of carbon were observed in co‐cultures of *C. vulgaris* and *Tistrella* sp. from day 3 of cultivation, which might have resulted from a release by dead microalgal cells. Alternatively, elevated carbon levels might have been formed by *Tistrella* sp., as bacteria were shown of being capable to release dissolved organic carbon into the environment in co‐culture with microalgae (Cho et al. 2015). Interestingly, ammonium levels stagnated upon the onset of *C. vulgaris* growth retardation. *Tistrella* sp. could have caused the observed increased ammonium levels in co‐culture, as bacteria affiliated with Rhodospirillaceae were reported of being involved in the fixation of nitrogen (Madigan et al. 1984). In contrast, dying microalgae being the main consumers of the ammonium could have caused the halt, which is in accordance with observations for co‐cultures conducted with *Pseudomonas* sp. and *Sphingopyxis* sp.

Microalgae exhibit extensive potential for counteracting imminent challenges in the nutraceuticals, pharmaceutical, and biomaterials sectors but lack in economic viability. Biotechnological systems for contamination control could advance the economic viability of microalgal feedstock, but the selection of suitable strains remains challenging. The study provides a selection of indigenous prokaryotes and eukaryotes for artificially tailoring microbial biocenoses that could benefit from stable associations in culture with *C. vulgaris*. Total diversity in phototrophic *C. vulgaris* cultures assessed by amplicon sequencing of cultures subjected to five different cultivation conditions showed 2 eukaryotic and 40 prokaryotic taxa to remain stably associated with *C. vulgaris* despite altering cultivation conditions. Controlled co‐cultures with indigenous prokaryotes showed stable associations of *C. vulgaris* with *Sphingopyxis* sp. and *Pseudomonas* sp., but impaired growth with *Tistrella* sp.. Out‐competition of *C. vulgaris* due to ammonium or phosphate limitation was not observed, despite significantly elevated growth of *Sphingopyxis* sp. (Wilcoxon rank‐sum test, W = 27, *P* < 0.05) and *Tistrella* sp. (Wilcoxon rank‐sum test, W = 18, *P* < 0.05).

Hence, the study provides a selection of stable indigenous prokaryotes and eukaryotes for artificially tailoring microbial biocenoses. Following a bottom‐up approach, it provides a base for controlled co‐cultures and thus the establishment of even more complex biocenoses using interkingdom assemblages. Such assemblages can benefit from functional richness for improved nutrient utilization, as well as bacterial load control, which can enhance microalgal feedstock production through improved culture stability and productivity.

Biotechnological systems for contamination control harnessing functionally rich interkingdom assemblages including prokaryotes and higher trophic levels, such as *Colpoda* sp. and *Paracercomonas* sp. provide potential for improving stability and productivity in microalgae cultures. They would not only add functional richness in terms of improving nutrient utilization, but could also contribute to bacterial load control, which can enhance microalgal feedstock production through improved culture stability and productivity. Despite the relevance of this study in the search of biocenoses allowing for stable microalgae cultivation conditions, the obtained results further lay the foundation for emerging processes, such as selective inactivation by pulsed electric field processing.

We gratefully acknowledge the support of the Genomic Diversity Centre at ETH Zürich and Silvia Kobel for their support with amplicon sequencing, as well as ScopeM for SEM imaging.

## Authors contributions

I.H. and A.M. conceptualized the study, I.H. developed the methodology, conducted laboratory experiments, and collected and visualized the data, J.C.W. developed the bioinformatics pipeline and conducted the analysis, H.H., S.B., and S.F. conducted cultivations in FPA, and provided *C. vulgaris* biomass and cultivation concepts, I.H., L.B., M.S., and A.M. developed the study layout; all authors contributed to analysis and writing of the manuscript.
